# Cellular and molecular mechanisms of hepatic ischemia-reperfusion injury: The role of oxidative stress and therapeutic approaches

**DOI:** 10.1016/j.redox.2024.103258

**Published:** 2024-06-27

**Authors:** Joseph George, Yongke Lu, Mutsumi Tsuchishima, Mikihiro Tsutsumi

**Affiliations:** a Department of Cancer Biology, Mayo Clinic, Jacksonville, FL, 32224, USA; b Department of Hepatology, Kanazawa Medical University, Uchinada, Ishikawa, 920-0293, Japan; c Center for Regenerative Medicine, Kanazawa Medical University Hospital, Uchinada, Ishikawa, 920-0293, Japan; d Department of Biomedical Sciences, Joan C. Edwards School of Medicine, Marshall University, Huntington, WV, 25755, USA

**Keywords:** Ischemia-reperfusion injury, Free radicals, Oxidative stress, Malondialdehyde, 4-Hydroxy-2-nonenal, γ-glutamyl transpeptidase

## Abstract

Ischemia-reperfusion (IR) or reoxygenation injury is the paradoxical exacerbation of cellular impairment following restoration of blood flow after a period of ischemia during surgical procedures or other conditions. Acute interruption of blood supply to the liver and subsequent reperfusion can result in hepatocyte injury, apoptosis, and necrosis. Since the liver requires a continuous supply of oxygen for many biochemical reactions, any obstruction of blood flow can rapidly lead to hepatic hypoxia, which could quickly progress to absolute anoxia. Reoxygenation results in the increased generation of reactive oxygen species and oxidative stress, which lead to the enhanced production of proinflammatory cytokines, chemokines, and other signaling molecules. Consequent acute inflammatory cascades lead to significant impairment of hepatocytes and nonparenchymal cells. Furthermore, the expression of several vascular growth factors results in the heterogeneous closure of numerous hepatic sinusoids, which leads to reduced oxygen supply in certain areas of the liver even after reperfusion. Therefore, it is vital to identify appropriate therapeutic modalities to mitigate hepatic IR injury and subsequent tissue damage. This review covers all the major aspects of cellular and molecular mechanisms underlying the pathogenesis of hepatic ischemia-reperfusion injury, with special emphasis on oxidative stress, associated inflammation and complications, and prospective therapeutic approaches.

## Graphical abstract

**Figure:**
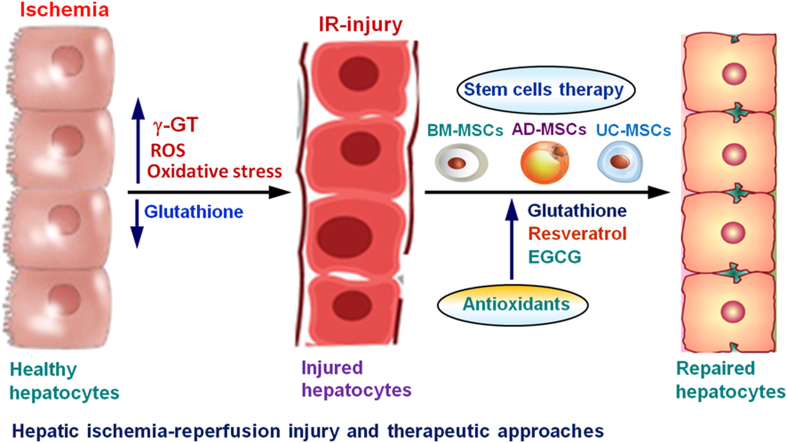

## Abbreviations

ALT: alanine transaminase
AST: aspartate transaminase
GGsTop: 2-amino-4{[3-(carboxymethyl) phenyl](methyl) phosphono} butanoic acid
γ-GT: γ-glutamyl transpeptidase
GSH: glutathione
HMGB1: high mobility group box 1
4-HNE: 4-hydroxy-2-nonenal
IR: ischemia-reperfusion
MDA: malondialdehyde
NASH: non-alcoholic steatohepatitis
ROS: reactive oxygen species

## 1 Facts

• Hepatic ischemia leads to cellular injury to hepatocytes and nonparenchymal cells.
• Reperfusion or restoration of blood supply during surgical procedures exacerbates ischemic tissue injury.
• Increased generation of reactive oxygen species and subsequent oxidative stress are responsible for cellular injury during ischemia and reperfusion.
• Elevated levels of γ-glutamyl transpeptidase activity and increased degradation of glutathione contribute to enhanced cellular oxidative stress.
• Expression of vascular growth factors and the resultant heterogeneous closure of hepatic sinusoids during ischemia exacerbate cellular injury and tissue damage.
• Inhibition of γ-glutamyl transpeptidase activity can decrease ischemia and reperfusion tissue damage.

## 2 Open questions

• How to prevent membrane lipid peroxidation and cellular injury during ischemia?
• Whether the use of potent antioxidants could significantly reduce ischemia and reperfusion cellular injury?
• How to store vital organs without tissue damage during transplantation procedures?

## 3 Introduction

Ischemia is a condition arising from reduced blood flow to tissues that results in decreased oxygen and nutrient supplies essential for normal cellular activities [1]. Blood flow can be blocked by thrombosis, embolism, or constriction of an artery [2]. Ischemia could also happen due to the narrowing or gradual thickening of the artery, as in atherosclerosis [3]. Furthermore, a trauma could lead to a significant ischemic brain injury that can result in cerebral edema [4]. Whatever be the reason, ischemic tissue injury is a major concern that requires firsthand medical intervention. Prolonged ischemia may lead to serious problems, including myocardial infarction, stroke, and other thrombotic processes, and also interfere with organ transplantation. Cellular ischemia follows a series of biochemical and morphological changes after about 10 min of injury, which may be reversed if blood supply could be rapidly restored to the injured cells.

## 4 Hepatic ischemia

A period of ischemia, or insufficient oxygen and blood supply, is necessary during many surgical procedures of the liver, especially on occasions while dealing with extensive hepatic trauma or transplantation [5]. The various other causes of hepatic ischemia are blood clots in the hepatic artery after a liver transplant, hepatic vasculitis, heat stroke, heart or respiratory failure, and chronic liver diseases. Whatever be the cause, hepatic ischemia could lead to injury to hepatocytes that results in impaired body metabolism. The liver receives around 25 % of the cardiac output, out of that 70 % through the portal vein, and 30 % from the hepatic artery [6]. Since the liver requires higher amount of oxygen for numerous biochemical reactions and metabolic processes, reduced blood supply can lead to immediate hepatic injury [7]. Hepatic ischemic injury is marked by a transient elevation of serum transaminases due to a decreased oxygen supply, increased oxidative stress, and associated cellular injury [6]. Resolution of the ischemia could restore the normal levels of serum transaminase within 3–5 days, depending on the condition.

## 5 Hepatic ischemia-reperfusion injury

The restoration of normal blood supply or reoxygenation of the tissue deprived of oxygen is termed reperfusion. However, this process will lead to additional complications as an “insult to injury” and is termed ischemia-reperfusion (IR) injury. It is a dynamic process involving two interrelated stages of ischemic insult and inflammation-mediated reperfusion injury [8]. The hepatic IR injury could also develop after organ transplantation, tumor resection or cardiopulmonary resuscitation, or as a result of trauma and shock [9]. The IR injury is mainly associated with enhanced production of free radicals and subsequent oxidative stress [10,11]. The extent of injury depends on how long the tissue was deprived of oxygen or blood supply. IR injury could lead to disseminated intravascular coagulation [12], hypovolemic shock [13], cardiac failure and arrest [14], increased toxicity of alcohol [15], and several other pathophysiological complications. Hepatic IR injury is a major clinical problem and could lead to multiple complications and poor outcome, including mortality after liver transplant surgery [16]. Currently, liver transplantation is the only effective approach to treat patients with terminal liver disease. However, liver dysfunction and failure are potential risks after transplantation and may affect patients’ survival and quality of life [17]. Therefore, it is important to investigate the molecular mechanisms involved in hepatic IR injury and the appropriate therapeutic modalities to arrest or attenuate the IR injury. Experimental rodent models of hepatic IR injury have provided significant information about the cellular and molecular mechanisms of hepatic IR injury and associated events [18,19]. However, the precise mechanisms of the pathogenesis of hepatic IR injury, the molecules involved, and the process of liver regeneration and repair after IR injury are still obscure. In this review, we delineate the underlying molecular mechanisms of hepatic IR injury employing existing knowledge and information, depict the downstream cascade associated with inflammation and complications, and describe the current treatment strategies and therapeutic approaches.

## 6 Parameters involved in hepatic ischemia-reperfusion injury

A variety of factors are involved in the development of hepatic IR injury, including activation of Kupffer cells, upregulation of pro-inflammatory cytokines, intracellular calcium overload, and oxidative stress, each contributing to the overall pathophysiology to varying degrees [20]. The formation of reactive oxygen species (ROS) and subsequent cellular oxidative stress are the most invoked mechanisms in the development of hepatic IR injury. Hepatic ischemia leads to tissue hypoxia, which in turn triggers hypoxia-inducible factors (HIFs) that regulate the expression of numerous genes involved in cell survival, angiogenesis, glycolysis, and cell invasion [21]. The decreased levels of oxygen and associated changes in metabolite levels can be sensed by several stress pathways that result in the generation of ROS by mitochondria. Subsequent reperfusion results in the activation of Kupffer cells, which in turn generate excessive amount of ROS such as superoxides, hydrogen peroxide, and hydroxyl radicals that lead to cellular impairment and hepatic inflammation [22]. The elevated levels of intracellular ROS lead to oxidative stress that causes damage to cellular lipids, proteins, and nucleic acids. Oxidative stress leads to enhanced peroxidation of membrane lipids that causes structural and functional impairment, leading to cell death [23]. Furthermore, pro-inflammatory cytokines, chemokines, and other inflammatory mediators produced by the damaged cells contribute to the systemic inflammatory syndrome and post-ischemic tissue injury [24]. The inflammatory mediators activate and drive neutrophils into the post-ischemic liver, which further increases ROS levels and triggers the synthesis of excessive proteases such as matrix metalloproteases [25]. Apart from the inflammatory responses, ROS induces the expression of endothelin-1, a potent endogenous vasoconstrictor mainly secreted by endothelial cells, which results in vasoconstriction of hepatic sinusoids [26,27]. This leads to the heterogeneous closure of numerous hepatic microvessels that results in reduced oxygen supply in certain areas of the liver even after reperfusion, leading to further complications [28].

## 7 Molecular mechanisms of ischemia-reperfusion injury

**Fig. 1:**
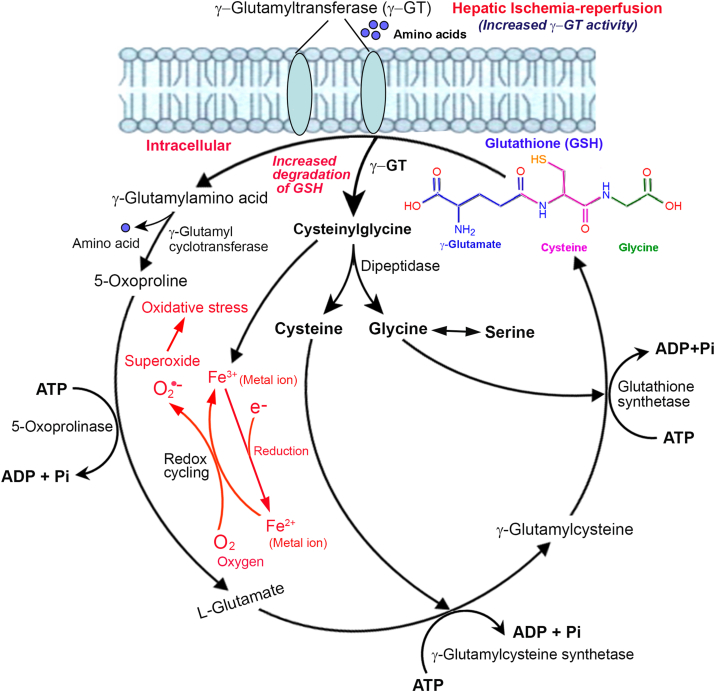
The γ−glutamyl cycle indicating elevated levels of γ-glutamyl activity and increased degradation of glutathione during hepatic ischemia-reperfusion. γ-Glutamyl transpeptidase (γ-GT) is present in the outer surface of the cell membrane and transfers the γ-glutamyl moiety of glutathione into glutamate and γ-glutamyl-amino acids with a byproduct cysteinylglycine. The cysteinylglycine is a highly reactive thiol compound and reduces Fe^3+^ (ferric iron) into Fe^2+^ (ferrous ion) with the donation of an electron. In the subsequent redox reaction, oxygen (O_2_) takes away an electron from Fe^2+^ and transform to Fe^3+^ with the generation of superoxide. Molecular oxygen (dioxygen) is a diradical containing two unpaired electrons, and superoxide is generated from the addition of an electron that fills one of the two degenerate molecular orbitals, leaving a charged ionic species with a single unpaired electron and a net negative charge of −1. The repeated redox cycling causes elevation of reactive oxygen species (ROS) which in turn results in intracellular oxidative stress.

A schematic diagram of the major molecular mechanisms involved in the generation of reactive oxygen species (ROS), oxidative stress, and the pathogenesis of liver injury during ischemia and reperfusion is presented in Fig. 1. The metabolic processes are mainly happening in hepatocytes. In addition, the impairment of first-line antioxidant defense mechanisms involving glutathione, glutathione peroxidase, superoxide dismutase, and catalase contributes to the pathogenesis of liver injury during ischemia and reperfusion [29].

### 7.1 Free radicals, reactive oxygen species, oxidative stress, and lipid peroxidation

The generation of free radicals and ROS is part of the regular biochemical and metabolic processes in biological systems, which are instantly removed under normal physiological conditions. Radicals (often called free radicals) are produced during a variety of biochemical reactions and also in pathogenic defense processes [30]. A free radical is an independent molecule with one or more unpaired electrons in its outermost atomic orbital. In a quest to fill the partially empty valence shell, the highly reactive free radicals “steal” electrons from the atoms of other molecules present in the cells and tissues. This electron-stealing frenzy produces oxidative damage to cell membranes, proteins, and DNA and is an underlying cause for many chronic diseases, including cardiovascular disease, diabetes, and cancer [31,32]. Reactive oxygen species are a subset of free radicals that contain a highly reactive oxygen molecule, such as superoxide (O_2_•−). Examples of ROS are peroxides, superoxide, hydroxyl radicals, singlet oxygen, and alpha-oxygen [33]. At normal physiological levels, ROS serve as key redox signaling agents for over 40 enzymes, most prominently NADPH oxidases (NOX) and the mitochondrial electron transport chain [34]. There are seven isoforms of NOX that have been characterized in humans, which include NOX1, NOX2, NOX3, NOX4, NOX5, DUOX1, and DUOX2 [35]. Each NOX isoform is differentiated by the specific catalytic subunit, interacting proteins, and subcellular localization. It was reported that remote ischemic preconditioning (IPC) has significantly reduced the IR-induced hepatic NOX2 expression, but the expression of NOX4 has been unchanged [36].

The term “oxidative stress” refers to elevated intracellular levels of ROS, which usually occur when there is an imbalance between the generation and removal of oxidative free radicals. Under normal physiological conditions, any excess amount of ROS generated during biochemical and metabolic processes will be instantly and quickly removed by the potent antioxidant system present in the body, and maintain a healthy balance between generation and disposal. However, during pathological conditions, the balance between ROS generation and removal will be impaired, which results in cellular oxidative stress that causes damage to cell membranes and macromolecules [37]. The term “lipid peroxidation” refers to the chain reactions of oxidative damage to membrane lipids during oxidative stress, leading to cell injury and ultimate cell death. It occurs when radicals “steal” electrons from the lipids of the cell membrane and induce a series of peroxide reactions of free fatty acids [38]. Cell membranes are highly sensitive to free radicals due to the presence of polyunsaturated fatty acids such as linoleic acid and arachidonic acid. Excessive production of ROS, decreased antioxidant defense system, increased cellular oxidative stress, and numerous downstream events are responsible for extensive liver injury and necrosis after ischemia-reperfusion [39].

### 7.2 Glutathione

Glutathione (GSH) is a tripeptide (Glu-Cys-Gly) composed of three amino acids: l-glutamate, l-cysteine, and l-glycine (Fig. 1). Glutamic acid is linked to cysteine through the γ-carboxyl group instead of the usual α-carboxyl group. Therefore, the bond is protected from common peptidases, and only the γ-glutamyl transpeptidase (γ-GT) can break it. Glutathione is a potent antioxidant that is involved in multiple cellular defense mechanisms, including cytokine production, cell proliferation, and immune responses [40]. Glutathione also involves regulation of metabolism and gene expression, signal transduction and apoptosis, and DNA and protein synthesis. The reduced form of glutathione (GSH) plays a pivotal role in removing free radicals from the liver and other vital organs. The sulfhydryl group (−SH) of the cysteine present in glutathione is involved in reduction and conjugation reactions and is responsible for the removal of peroxides and multiple xenobiotic compounds [41]. The intracellular concentration of GSH in hepatocytes is up to 10-fold higher than extracellular GSH. During ischemia, the intracellular GSH will be flushed to extracellular space in order to scavenge free radicals, which results in the depletion of intracellular GSH. A significant decrease in hepatic GSH level was reported during ischemia and also at 2 h after reperfusion [42]. We have noticed a marked decrease in hepatic GSH content during IR injury in rats [18].

### 7.3 γ-Glutamyl transpeptidase

Gamma-glutamyl transpeptidase (γ-GT), also named γ-glutamyl transferase or γ-glutamyl peptidyltransferase, is a heterodimeric enzyme present in most organisms ranging from bacteria to mammals [43]. The mammalian γ-GT is present on the outer surface of the plasma membrane, anchoring its N-terminal to the membrane, and exhibits secretory or absorptive functions (Fig. 1) [44]. The enzyme γ-GT plays a prominent role in glutathione metabolism and involves the transfer of amino acids across the cell membrane. It catalyzes the transfer of the γ-glutamyl group of glutathione and other γ-glutamyl amides to water (hydrolysis) or amino acids and peptides (transpeptidation) [45]. γ-GT plays a pivotal role in antioxidant defense and xenobiotic metabolism and is also associated with numerous pathological conditions, including cardiovascular diseases, liver disease, and cancer [46,47]. Ischemia-reperfusion injury leads to extreme hepatic necrosis that results in leakage of γ-GT into the blood stream and subsequent elevation of serum γ-GT levels [48]. Serum γ-GT has been widely used as an index of liver dysfunction, fatty liver disease, and a marker of alcohol abuse [49].

In mammalian cells, glutathione degradation occurs exclusively in the extracellular space [50]. The extracellular γ-GT catalyzes the first step in GSH degradation and transfers the γ-glutamyl moiety of GSH to an amino acid to form γ-glutamylamino acid with a byproduct of cysteinylglycine [51]. The enzyme γ-glutamyl cyclotransferase cleaves γ-glutamylamino acid to an amino acid and 5-oxoproline. The latter transforms into l-glutamate and enters the glutathione cycle (Fig. 1). Cysteinylglycine is further cleaved by a dipeptidase into cysteine and glycine, which are utilized in the biosynthesis of GSH. Cysteinylglycine is a highly reactive thiol compound that has very high physiological activity. It can reduce oxygen under normal physiological conditions by reducing a metal ion or similar compounds [52]. This process is known as redox-cycling and produces superoxide, which results in increased cellular oxidative stress that subsequently triggers oxidative reactions of biomembrane lipids and other macromolecules (Fig. 1) [53]. During IR injury, extreme cellular necrosis occurs, which results in the release of membrane bound γ-GT into the extracellular space. This leads to enhanced degradation of GSH driven by γ-GT, which in turn generates increased ROS and oxidative stress. This will further exacerbate the situation and induce additional membrane injury and cellular impairment. As an “insult to injury,” the restoration of blood supply or reperfusion of ischemic tissue leads to increased injury, mainly due to enhanced production of free radicals [18]. Glutathione driven oxidative damage generated by γ-GT could produce preneoplastic foci in the liver and may lead to hepatocarcinogenesis [52].

### 7.4 Mechanism of increased generation of reactive oxygen species and development of oxidative stress during ischemia and reperfusion

The molecular mechanism of increased generation of reactive oxygen species and elevation of oxidative stress during IR injury is depicted in Fig. 1. There will be increased activity of γ-GT and enhanced degradation of GSH during ischemia and reperfusion, which results in elevated levels of cysteinylglycine. As discussed above, cysteinylglycine is a highly reactive thiol compound that can reduce Fe^3+^ into Fe^2+^ with the donation of an electron. In the subsequent redox reaction, oxygen (O_2_) takes away an electron from Fe^2+^ and transforms it to Fe^3+^ with the generation of superoxide.

Fe^2+^ + O_2_ ←→ [Fe^2+^−O_2_ ←→ Fe^3+^−O_2_•−] ←→ Fe^3+^ + O_2_•− (superoxide) → cellular injury

Molecular oxygen is a diradical that consists of two unpaired electrons. Superoxide is generated when an electron is added that fills one of the two degenerate molecular orbitals, leaving a charged ionic species with a single unpaired electron and a net negative charge of −1. In the reverse reaction, an electron from cysteinylglycine converts Fe^3+^ back to Fe^2+^ and the process continues. This redox cycling refers to the ability to cycle between oxidized and reduced forms, and the process results in the production of ROS, such as superoxide. The repeated redox cycling causes elevation of free radicals (ROS), which in turn results in intracellular oxidative stress (Fig. 1). Under normal conditions, the enzyme superoxide dismutase (SOD) quickly catalyzes the dismutation of superoxide (O_2_•−) along with water (H_2_O) into ordinary molecular oxygen (O_2_) and hydrogen peroxide (H_2_O_2_) [54]. The H_2_O_2_ thus formed is highly reactive and harmful to cell membranes and other molecules. It is quickly degraded by the ubiquitous enzyme catalase into water and molecular oxygen (2H_2_O_2_ → 2H_2_O + O_2_) [55]. However, during ischemia and reperfusion, there is increased activity of γ-GT and enhanced degradation of glutathione with the net result of elevated superoxide levels. On the other hand, there is a decreased activity of the free radical scavengers SOD and catalase due to increased cellular injury. This will exacerbate membrane lipid peroxidation and also affect other vital molecules such as proteins and nucleic acids. Furthermore, during reperfusion, multiple free radicals and nitric oxide (NO) are formed, which cause additional inflammation, membrane lipid peroxidation, and apoptosis that aggravate the situation [56].

### 7.5 Role of mitochondrial ROS in the pathogenesis of ischemia-reperfusion injury

It has been well documented that during ischemia and reperfusion, the extensive generation of ROS by mitochondria plays a critical role in the impairment of cellular components and triggers cell death [57]. Under normal physiological conditions, the electron transport chain of mitochondria produces a moderate quantity of ROS, which will be removed by the potent antioxidant system, and the balance is maintained. However, during ischemia, mitochondria generate large amounts of ROS to maintain the redox balance, which results in the increased utilization of endogenous antioxidants [58]. When the balance between the increased amount of ROS and the antioxidant system scavenging system is impaired, ROS accumulates in the mitochondria, leading to oxidative stress, inflammation, cell death, and organ failure [59]. The ROS generated inside mitochondria plays a prominent role in contributing to IR injury, inducing mitochondrial permeability transition and oxidative damage to mitochondrial structures and molecules [60]. The onset of ischemia triggers impairment of electron transport and the oxidative phosphorylation system of mitochondria, which leads to structural and functional deterioration of the mitochondrial membrane. Since mitochondria are the battery of the cell, damage to the mitochondrial membrane results in mitochondrial dysfunction and the collapse of multiple cellular signaling pathways, leading to necrosis and cell death. Overall, the ROS formed within mitochondria play a significant role in the deleterious cascade of events associated with hepatic IR injury, which might be a potent therapeutic target to prevent adverse events during liver transplant surgery.

## 8 Downstream effects of oxidative stress during ischemia and reperfusion

The increased oxidative stress that develops during ischemia and reperfusion is the major reason for cellular injury during hepatic surgery or transplantation. Hypoxia alters the mitochondrial electron transport chain and contributes to the development of enhanced ROS in hepatic ischemia [61]. The increased ROS during ischemia and reperfusion may lead to a series of processes in the liver leading to hepatic injury, cellular necrosis, apoptosis, and cell death.

### 8.1 Translocation of nuclear protein HMGB1

**Fig. 2:**
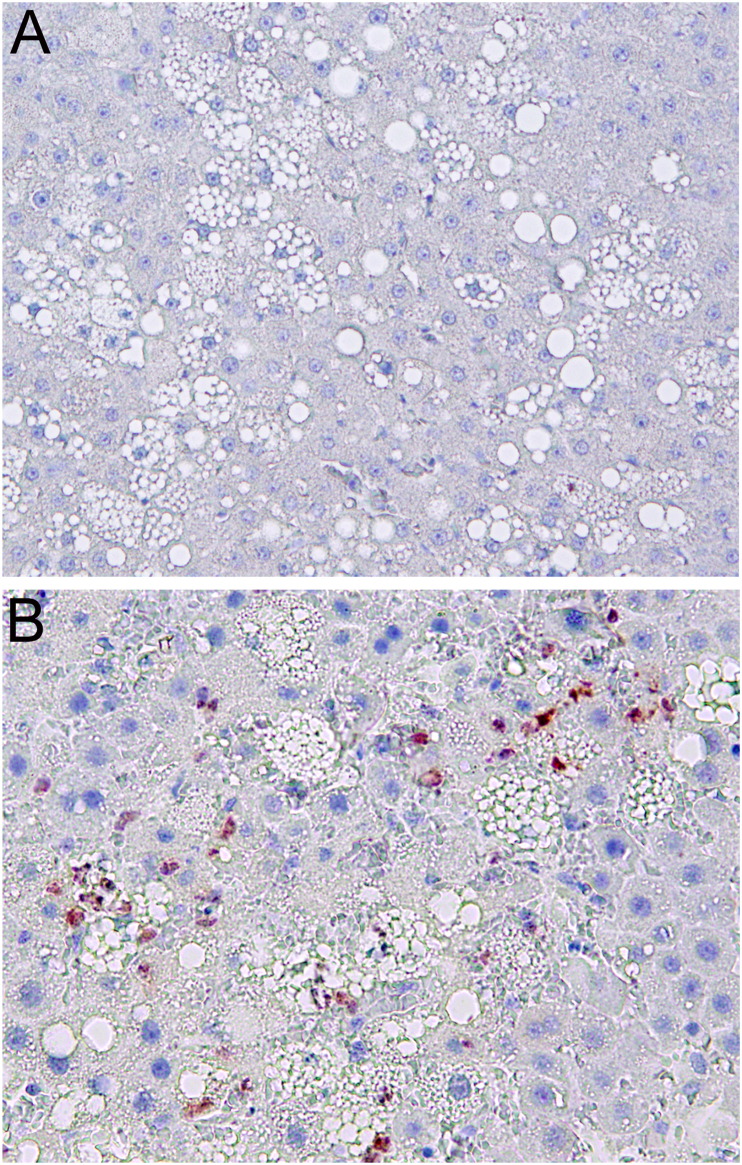
Immunohistochemical staining for high mobility group box 1 (HMGB1) (antibody used (GeneTex Cat# GTX127344, RRID:AB_11164700): in rat liver with hepatic steatosis. **(A)** Control liver showing the complete absence of HMGB1 staining. **(B)** Marked and prominent staining of HMGB1 after ischemia-reperfusion in the necrotic zone as a response to hepatic inflammation (x200). HMGB1 is a nuclear protein that translocates to the cytoplasm and extracellular compartments during ischemia-reperfusion injury. Tissue oxidative stress is a major factor that induces the secretion of HMGB1 from the nucleus and its relocation to the extracellular matrix to perform pivotal roles in the regulation of cellular response to inflammation. *(Originally published as a Figure panel in Br J Pharmacol 2020; 177: 5195–*5207 by *the authors).*

High mobility group box 1 (HMGB1) is a highly conserved nuclear protein that is released during cellular injury, translocates to the extracellular space, and acts as a chemokine [62]. HMGB1 is involved in DNA repair, regulates transcription, promotes the secretion of cytokines, and potentially contributes to tissue repair and regeneration [63]. HMGB1 is actively secreted from a variety of immune and non-immune cells, including hepatocytes, in response to various stimuli, such as proinflammatory cytokines [64]. Cellular oxidative stress is one of the key factors that induces the secretion of HMGB1 from the nucleus and its translocation to the extracellular space to perform important roles in the regulation of cellular responses to inflammation and injury [65]. As a representative damage-associated molecular pattern (DAMP), HMGB1 normally presents inside cells but can be secreted into the extracellular space through passive or active release [66]. Upon translocation to the extracellular space, HMGB1 forms complexes with central mediators of inflammation such as TLR4, CXCL12, and RAGE, with subsequent recruitment of inflammatory cells and production of cytokines [66]. Fig. 2 demonstrates marked staining of HMGB1 in the extracellular compartment of the rat liver after IR injury. It was observed that selective IR injury induces HMGB1 translocation to the adjacent non-ischemic hepatic lobes also [67]. Therefore, targeting HMGB1 with suitable antioxidants could be an appropriate therapeutic method for inflammation-associated diseases such as ischemia and reperfusion injury, arthritis, diabetes, and cancer.

It was reported that inflammasome-mediated inflammation plays a significant role in the pathogenesis of hepatic IR injury [68]. Inflammasomes are a type of intracellular multimolecular complex that actively participates in innate immune responses and proinflammatory signaling pathways [69]. Among them, the cytokine-producing nucleotide-binding domain leucine-rich repeat and pyrin-containing receptor 3 (NLRP3) inflammasome play a central role in the immune response to various pathogen-derived as well as danger-associated signals [69]. NLRP3 pathways can be enhanced under oxidative stress conditions and are subject to redox regulation [70]. NLRP3 is highly expressed in liver macrophages, and the assembly of the NLRP3 inflammasome could lead to hepatic IR injury and thus promise as a therapeutic target.

### 8.2 Increased release of tumor necrosis factor

**Fig. 3:**
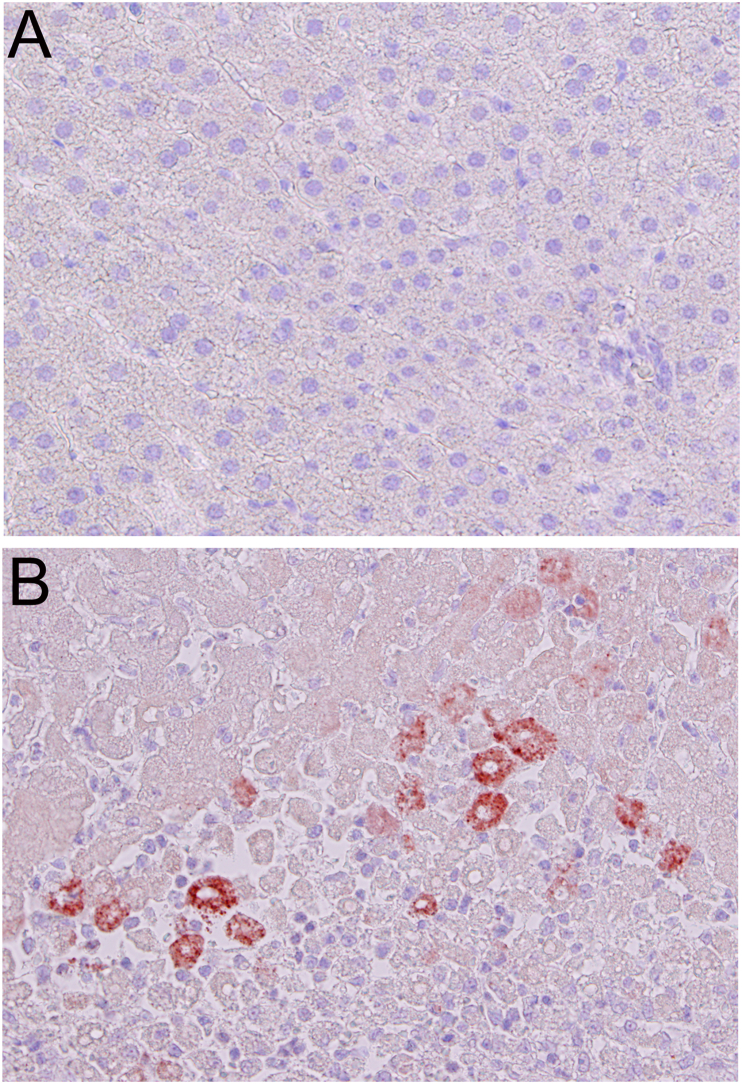
Immunohistochemical staining for tumor necrosis factor-α (TNF-α) (antibody used: Cat# ab6671, Abcam, Tokyo, Japan) in rat liver sections after ischemia-reperfusion injury. **(A)** Control liver showing complete absence of TNF-α staining. **(B)** Intense and strong staining of TNF-α in macrophages that infiltrated into the necrotic zone (x100). TNF-α is a pro-inflammatory cytokine produced by activated macrophages during acute inflammation and serves as a marker for the degree of hepatic injury during ischemia-reperfusion. *(Originally published as a Figure panel in Am J Physiol Gastrointest Liver Physiol 2016; 311: G305–G3*12 by *the authors).*

Tumor necrosis factor (TNF, cachexin, or cachectin; formerly known as tumor necrosis factor alpha, or TNF-α) is a potent pro-inflammatory cytokine that has a major role in cell signaling and the formation of acute phase reactions [71]. It is mainly released by activated macrophages during infection or inflammation to alert other cells in the immune system as part of an inflammatory response. In the liver, TNF induces numerous biological and pathological responses such as hepatocyte apoptosis and necrosis, liver inflammation and regeneration, autoimmunity, and progression to hepatocellular carcinoma [72]. The rate of expression of TNF serves as a marker for the degree of hepatic inflammation. Fig. 3 demonstrates intense and strong staining of TNF in macrophages that infiltrated into the necrotic zone during IR injury in rat livers. Cellular oxidative stress is a major factor that triggers inflammatory signal to macrophages, which in turn releases TNF in several hepatic pathological conditions such as ischemia, NASH, and hepatic fibrosis [18,73]. Targeting TNF-α and its receptors may be a potent therapeutic strategy to arrest excessive inflammatory responses in IR injury and several autoimmune diseases.

### 8.3 Enhanced rate of membrane lipid peroxidation

Lipid peroxidation refers to the chain of reactions involved in the oxidative degradation of membrane lipids in biological systems. During this process, free radicals “steal” electrons from the hydrogen atom of lipids present in cell membranes as the first phase. Cell membranes are highly sensitive to lipid peroxidation due to the extensive presence of polyunsaturated fatty acids that contain multiple double bonds of the methylene group (-CH2) with reactive hydrogen atoms [74]. The radical formed in the first phase reacts with oxygen and forms a peroxyl radical that further reacts with adjacent polyunsaturated fatty acids to form a hydroperoxide and an alkyl radical, thus forming a chain of reactions that causes damage to the cell membrane. Increased generation of free radicals and cellular oxidative stress is an integral part of the pathogenesis of almost all liver diseases, including NASH and hepatic fibrosis [75,76]. The elevated levels of γ-GT and increased production of ROS during IR injury could result in extensive membrane lipid peroxidation, leading to apoptosis or necrosis. Increased oxidative stress and accompanying lipid peroxidation are major factors involved in aging and associated disorders [77].

**Fig. 4:**
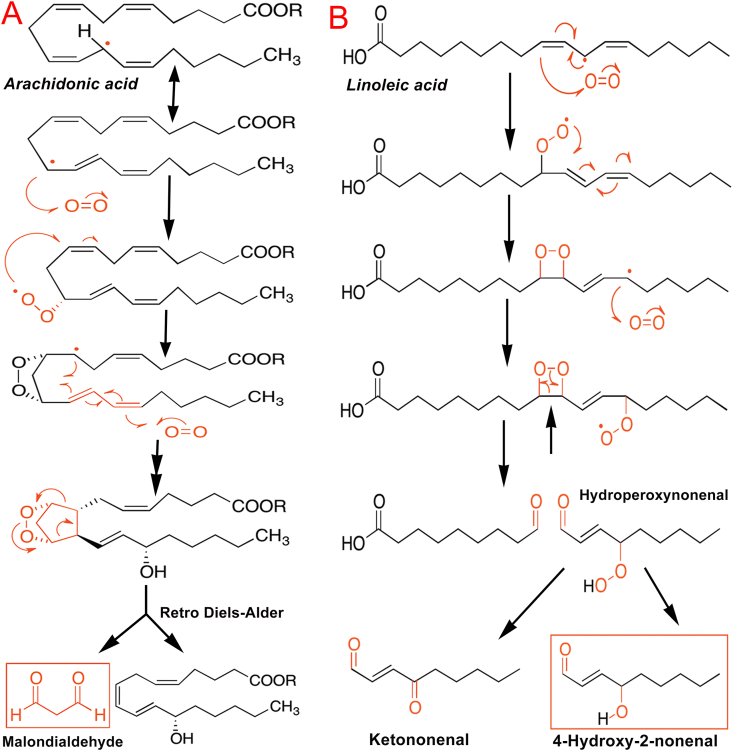
Lipid peroxidation of polyunsaturated fatty acids depicting the production of toxic end products malondialdehyde (MDA) and 4-hydroxy-2-nonenal (4-HNE). The toxic aldehydes MDA and 4-HNE can react with the -NH2 group of proteins and DNA bases to form adducts that can cause mutations. **(A)** Oxidative degradation of arachidonic acid and formation of MDA. **(B)** Oxidative breakdown of linoleic acid to hydroperoxynonenal and then to hydroxynonenal.

Malondialdehyde (MDA) and 4-hydroxy-2-nonenal (4-HNE) are the two major end products and tissue markers of lipid peroxidation. Malondialdehyde is considered the most mutagenic product of lipid peroxidation, while 4-HNE is the most toxic. MDA is a highly reactive three-carbon dialdehyde formed as an end product of arachidonic acid and large polyunsaturated fatty acid metabolism [78]. The oxidative degradation of an arachidonic acid ester molecule and the formation of MDA are presented in Fig. 4A. In addition to hydroperoxides, the peroxidation of arachidonic acid can generate cyclic peroxides such as isoprostanoids [79]. On the other hand, they cycle together with the addition of a second oxygen molecule. These intermediates generate MDA through a retro-Diels–Alder reaction (Fig. 4A). MDA can readily bind to several functional groups on proteins and nucleic acids and form adducts. MDA adducts can participate in secondary deleterious reactions through intermolecular and intramolecular protein/DNA crosslinking that may cause profound alteration in the biochemical properties of macromolecules. We have reported remarkably increased levels of MDA in the hepatic tissue during experimentally induced ischemia-reperfusion injury in rat livers [18,39,48].

The highly reactive lipid peroxidation end product, 4-HNE is an α,β-unsaturated aldehyde formed by the peroxidation of omega-6 unsaturated fatty acids such as linoleic acid [80]. As a highly reactive aldehyde, 4-HNE can disrupt signal transduction and protein activity, induce inflammation, and trigger cellular apoptosis. It can efficiently react with sulfhydryl groups or histidine and lysine groups of proteins to form stable HNE-protein adducts, in addition to phospholipids and nucleic acids [81]. The oxidative degradation of linoleic acid, depicting the production of 4-HNE is presented in Fig. 4B. The formation of 4-HNE is a reliable biomarker for the oxidative degradation of membrane lipids and is directly correlated with cellular oxidative stress. Increased lipid peroxidation and the formation of 4-HNE-protein adducts have been reported after ischemia and reperfusion during the transplantation of rat hearts [82]. Increased tissue levels of 4-HNE is linked with a large number of pathological conditions, including Alzheimer's disease, diabetes, cataract, atherosclerosis, fatty liver diseases, fibrogenesis, and cancer [83].

## 9 Current therapeutic approaches to arrest ischemia-reperfusion injury

**Fig. 5:**
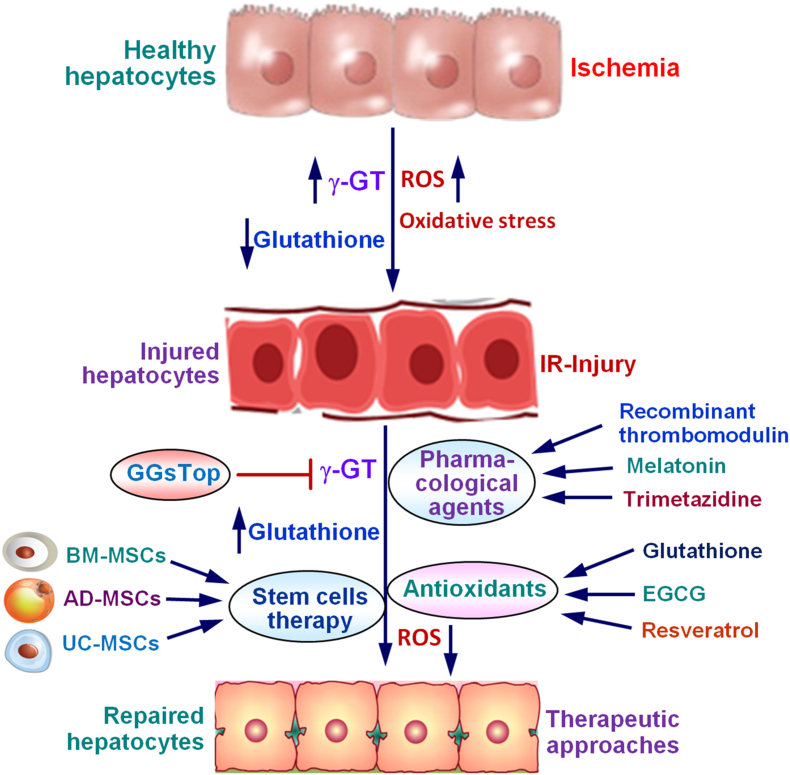
Schematic presentation of the prospective therapeutic approaches to attenuate ischemia-reperfusion injury, promote repair of impaired hepatocytes, and regenerate the injured liver tissue. Various pharmacological agents, potent antioxidants, and mesenchymal stem cells from different sources could be used for the purpose.

Since the pathophysiology of hepatic IR injury involves multiple molecular mechanisms, various types of pharmacological interventions, antioxidant therapy, and stem cell therapy have been tested to suppress the phenomenon. However, many of these strategies are currently at the stage of animal experimental models only. The prospective therapeutic approaches to arrest ischemia-reperfusion injury are depicted in Fig. 5.

### 9.1 Inhibition of γ-glutamyl transpeptidase

As described in Fig. 1 and the associated text, there is increased activity of γ-GT and enhanced degradation of GSH, which lead to elevated oxidative stress and lipid peroxidation during hepatic IR injury. Therefore, inhibition of γ-GT is an effective measure to arrest increased production of ROS, subsequent oxidative stress, and the associated lipid peroxidation. We have demonstrated that intravenous injection of GGsTop is an effective method to inhibit γ-GT and prevent hepatic IR injury in rat models [18,48]. GGsTop (2-amino-4{[3-(carboxymethyl) phenyl](methyl) phosphono} butanoic acid) is a novel phosphonate and a potent irreversible inhibitor of γ-GT [84]. GGsTop specifically inhibits human γ-GT more than 100-fold compared to acivicin and does not affect glutamine amidotransferase [85]. GGsTop covalently binds between the side chain oxygen of Thr-381 of human gamma GT1 (hGGT1) and the phosphate of GGsTop resulting in an enzyme-inhibitor complex [86]. Intravenous administration of GGsTop at a single dose of 30 or 100 mg/kg body weight in rats did not produce abnormalities in behavior, body weight, or amount of food intake for 2 weeks [87]. Besides, GGsTop exhibited no cytotoxicity towards human fibroblasts and hepatic stellate cells up to 1 mM concentration in culture [88]. Furthermore, it was reported that enhanced γ-GT activity contributes to cardiac impairment after myocardial ischemia/reperfusion through oxidative stress, and treatment with GGsTop has potential therapeutic implications to prevent myocardial ischemia/reperfusion injury [89]. Therefore, treatment with GGsTop could be an appropriate therapeutic method to reduce ischemia/reperfusion-induced liver injury during surgical resection and transplantation.

### 9.2 Use of other pharmacological agents

#### 9.2.1 Recombinant thrombomodulin

Many pharmacological agents have been reported that could alleviate or arrest hepatic IR injury during resection or transplantation. We have observed that intravenous injection of recombinant thrombomodulin (rTM) can significantly reduce the expression of TNF-α and the formation of 4-HNE during experimentally induced hepatic IR injury [39]. Thrombomodulin (TM), or human CD141+ is a 74 kDa glycoprotein that expresses on the surface of endothelial cells and serves as a cofactor for thrombin-mediated activation of protein C [90,91]. Recombinant thrombomodulin is a novel physiologic anticoagulant protein used to treat sepsis-induced disseminated intravascular coagulation (DIC). It is composed of the active extracellular domain of thrombomodulin that could serve the same as thrombomodulin and also be an anti-inflammatory agent [92]. As in the case of thrombomodulin, rTM accelerates the thrombin-catalyzed conversion of protein C to activated protein C that inhibits monocyte and macrophage activation [93]. It was reported that rTM could ameliorate autoimmune vasculitis through a combination of immune response regulation and tissue injury protection [94]. Besides, rTM also exhibits an anti-inflammatory effect through binding and inhibiting the proinflammatory mediator, high mobility group box 1 (HMGB1) protein, which in turn leads to reduction of TNF-α and other inflammatory cytokines [95,96]. It was reported that rTM ameliorates hepatic IR injury in a toll-like receptor-4 (TLR-4) pathway dependent manner and is suggested as a novel medicine for liver transplantation [96]. Therefore, rTM might be a useful agent to suppress hepatic inflammation and the associated IR injury during surgical procedures.

#### 9.2.2 Melatonin

Melatonin is a potent antioxidant and a hormone synthesized in the pineal gland at night that controls the sleep-wake cycle in vertebrates [97]. Melatonin acts as a direct scavenger of free radicals and ROS, including OH•, O_2_•−, and the reactive nitrogen species NO• [98]. Through its receptors, melatonin stimulates various potent antioxidant enzymes that include superoxide dismutase, catalase, and glutathione peroxidase to attenuate hepatic IR injury [99]. Several studies demonstrated that melatonin attenuates hepatic inflammation and IR injury by ensuring the synthesis of ATP in the liver, maintaining the stability of the mitochondrial membrane, and improving bile production [100,101]. It was shown that melatonin could decrease the activity of the nuclear factor kappa B (NF-κB) signaling pathway during hepatic IR injury, ameliorate the inflammatory response, maintain liver function, and increase the survival rate [102]. Furthermore, it was reported that melatonin protects the liver against IR injury through hemeoxygenase-1 induction that suppresses the type 1 interferon signaling pathway downstream of toll-like receptor 4 [103]. Overall, it is evident that melatonin could be used as a successful pharmacological agent to suppress IR injury during liver transplantation and associated procedures.

#### 9.2.3 Trimetazidine

Trimetazidine is a metabolic altering agent that inhibits the oxidation of fatty acids and improves myocardial glucose utilization. It is the first cytoprotective agent introduced against hepatic ischemic injury. It was observed that pretreatment of rats with trimetazidine prevented the deleterious effects of ischemia-reperfusion both at the cellular and mitochondrial levels in a dose-dependent manner [104]. Furthermore, it was reported that repeated administration of trimetazidine for 3 days was more effective than a single dose to protect the rat liver against IR-induced apoptosis and lipid peroxidation [105]. In addition, there was a significant increase in phosphorylated adenosine monophosphate-activated protein kinase (*p*-AMPK) and endothelial nitric oxide synthase (eNOS) levels in trimetazidine administered to rats for 3 days compared to the single dose. Trimetazidine inhibits β-oxidation of fatty acids by blocking long-chain 3-ketoacyl-CoA thiolase, which enhances glucose oxidation [106]. During ischemia, the energy obtained during glucose oxidation requires less oxygen consumption than in the β-oxidation process, which could maintain proper energy metabolism.

### 9.3 Antioxidants

Since excessive formation of ROS and subsequent oxidative stress are the major causes of the pathogenesis of IR injury, antioxidant therapy is one of the best options to attenuate hepatic IR injury during liver transplantation and associated procedures [107]. Under normal physiological conditions, the human body has well-developed antioxidant defense mechanisms to protect against oxidative impairment. However, during pathological or ischemic conditions, the antioxidant defense mechanisms will be impaired due to excessive production of free radicals and cellular oxidative stress. Glutathione is the most potent and powerful natural antioxidant present in all living organisms. It was reported that intravenous administration of glutathione during ischemia-reperfusion in rat livers significantly prevented hepatocyte necrosis with a 50–60 % decrease in AST and ALT levels [108]. Another potent and powerful natural antioxidant present in green tea is epigallocatechin-3-gallate (EGCG), which is a polyphenol catechin with eight hydroxyl groups (−OH) that are important for the antioxidant activities to bind and detoxify free radicals [109,110]. It was demonstrated that EGCG treatment attenuated hepatic IR injury by reducing oxidative stress and cellular apoptosis [111]. Resveratrol is a natural phenol abundant in red wine and berries that has potent antioxidant properties. Resveratrol has protective effects against cellular oxidative damage and hepatic IR injury through inhibition of endothelin-1 by suppressing the extracellular signal-regulated kinase (ERK) signaling pathway [112]. Genistein is another potent antioxidant that involves in multiple biochemical reactions [113]. It has been suggested that genistein might protect liver from IR injury during transplant surgery [114]. Even though antioxidant therapy may not be a powerful method to prevent IR injury during liver resection and transplant, it could definitely reduce the extent of hepatocyte injury and tissue damage.

### 9.4 Stem cells therapy

Mesenchymal stem cells (MSCs) are multipotent stromal cells that have the ability to penetrate into injured tissues, differentiate and multiply into specific cells, and induce pleiotropic signaling [115]. The new mass of differentiated cells could suppress tissue injury and restore normal functions impaired during ischemia-reperfusion. Several studies have demonstrated that MSCs derived from bone marrow, umbilical cord, or adipose tissue could attenuate hepatic IR injury through suppression of oxidative stress, inhibition of apoptosis, or immunomodulation [115,116]. Various autocrine and paracrine factors derived from the stromal cells seem to serve as reparative functions in the attenuation of hepatic IR injury. It was reported that transplantation of bone marrow-derived MSCs (BM-MSCs) ameliorates hepatic IR injury through inhibition of apoptosis and regeneration of hepatocytes in a rat model [117]. In addition, it was shown that systemic administration of adipose tissue-derived MSCs (AD-MSCs) maintained hepatocyte integrity and suppressed inflammatory responses, oxidative stress, and apoptosis in a rodent model of hepatic IR injury [118]. Furthermore, treatment with human umbilical cord-derived MSCs (UC-MSCs) prevented neutrophil infiltration, hepatocyte apoptosis, and expression of genes associated with inflammation and attenuated hepatic IR injury in a rat model [119]. It was observed that extracellular vesicles (EVs) derived from mouse bone marrow MSCs as well as EVs from human umbilical cord MSCs ameliorate hepatic IR injury by suppressing oxidative stress and modulating neutrophil inflammatory responses [120,121]. It was also noticed that portal vein administration of EVs derived from rat adipose tissue stem cells attenuated hepatic IR injury through activation of ERK1/2 and inactivation of glycogen synthase kinase-3 β (GSK-3β) signaling pathways [122]. The data from these studies indicate that MSCs and EVs derived from various sources could be effectively used as a therapeutic method to attenuate hepatic IR injury during liver transplantation and associated procedures.

## 10 Conclusions

Hepatic IR injury is a pathophysiological process resulting from ischemia-mediated cellular impairment that exacerbates upon reperfusion. Excessive formation of free radicals and subsequent oxidative stress during ischemia and after reperfusion are the major causes of IR injury. The resultant acute inflammatory cascade leads to significant impairment of hepatocytes and nonparenchymal cells, leading to lipid peroxidation, apoptosis, and hepatic necrosis. Multiple levels of pharmacological interventions, antioxidant therapy, and stem cell therapy have been tried to attenuate IR injury. Since oxidative stress is the major culprit in the pathogenesis of IR injury, antioxidant therapy is one of the best options to suppress the process. Recent studies indicated that mesenchymal stem cells and exosomes derived from various sources may be successfully used as a therapeutic method to alleviate hepatic IR injury during liver transplantation and associated procedures.

## Funding

This research work was supported by Grant from 10.13039/501100004043Kanazawa Medical University (Grant #RP 2020–03) to M. Tsutsumi.

## CRediT authorship contribution statement

**Joseph George:** Writing – review & editing, Writing – original draft, Validation, Resources, Methodology, Investigation. **Yongke Lu:** Writing – review & editing, Validation, Methodology, Formal analysis. **Mutsumi Tsuchishima:** Supervision, Investigation, Formal analysis, Conceptualization. **Mikihiro Tsutsumi:** Writing – review & editing, Supervision, Funding acquisition, Conceptualization.

## Declaration of competing interest

The authors declare that they have no known competing financial interests or personal relationships that could have appeared to influence the work reported in this paper.

## Data Availability

Data will be made available on request.
